# Climate change is associated with asynchrony in arrival between two sympatric cuckoos and both host arrival and prey emergence

**DOI:** 10.1098/rsos.231691

**Published:** 2024-01-17

**Authors:** Peter Mikula, Oleg V. Askeyev, Arthur O. Askeyev, Igor V. Askeyev, Federico Morelli, Annette Menzel, Piotr Tryjanowski

**Affiliations:** ^1^ TUM School of Life Sciences, Ecoclimatology, Technical University of Munich, 85354 Freising, Germany; ^2^ Institute for Advanced Study, Technical University of Munich, 85748 Garching, Germany; ^3^ Faculty of Environmental Sciences, Czech University of Life Sciences Prague, Kamýcká 129, 16500 Prague, Czech Republic; ^4^ Biomonitoring Laboratory, Institute of Problems in Ecology and Mineral Wealth, Tatarstan Academy of Sciences, Daurskaya Strasse 28, Kazan 420087, Tatarstan Republic, Russia; ^5^ Institute of Zoology, Poznań University of Life Sciences, Wojska Polskiego 71 C, 60-625 Poznań, Poland

**Keywords:** brood parasite, cuckoo, phenological mismatch, phenology, temperature, temporal trends

## Abstract

Matching the timing of spring arrival to the breeding grounds with hosts and prey is crucial for migratory brood parasites such as cuckoos. Previous studies have focused mostly on phenological mismatch between a single cuckoo species and its hosts but information regarding climate-driven mismatch between multiple sympatric cuckoo species and their hosts and invertebrate prey is still lacking. Here, we analysed long-term data (1988–2023) on the first arrival date of two declining migratory cuckoo species and their 14 migratory host species breeding in sympatry and prey emergence date in Tatarstan (southeast Russia). We found that the common cuckoo (*Cuculus canorus*; wintering in Africa) generally arrived on breeding grounds earlier than the oriental cuckoo (*Cuculus optatus*; wintering in southeast Asia and Australia). Both cuckoos have advanced their arrival dates over 36 years but less than their hosts, potentially resulting in an increasing arrival mismatch between cuckoos and their hosts. Moreover, cuckoo arrival advanced less than the emergence date of their prey over time. These observations indicate that climate change may disrupt co-fluctuation in the phenology of important life stages between multiple sympatric brood parasites, their hosts and prey with potential cascading consequences for population dynamics of involved species.

## 1. Introduction

Global climate change significantly affects the phenology, i.e. annual timing of recurring biological life cycle events, in many organisms, such as leafing and flowering in plants, and reproduction, migration and emergence in animals [1–6]. The timing of when migrant birds return to their breeding grounds is a key component of studies of the impact of climate change on bird populations. Many migratory bird species breeding in mid- and high latitudes migrate and breed earlier, specifically as a response to the higher spring temperatures and earlier peaks in their food abundance [4,7–9].

Rapid shifts in the abiotic environment driven by climate change do not affect all organisms at the same rate and level, potentially causing a mismatch in the phenology between organisms with different traits and positions in ecological networks [1]. Moreover, climate change may be less pronounced in some regions than others, resulting in temporally and spatially uneven phenological change [10]. The ecological consequences of climate-driven phenological mismatch have been primarily investigated in trophic webs [11,12]. However, differential phenological responses of organisms can likely also affect other ecological interactions, such as brood parasites and their hosts [13,14]. Migratory brood parasites need to synchronize their arrival not only with food availability but also with periods optimal for the exploitation of their hosts. However, differential responses to climate change may disrupt these synchronizations and may have contributed to population declines of some brood parasites [14,15]. For example, long-distance migratory brood parasitic birds may desynchronize their arrivals from those of their shorter-distance migratory hosts and emergence of non-migratory local prey at breeding grounds. This is because long-distance migratory brood parasites may rely more on circannual and endogenous mechanisms to time their migration rather than external cues, which are potentially tracked more by short-distance migrants or non-migratory prey [16,17].

The cuckoos (Cuculidae) are among the most famous obligate avian brood parasites [18,19]. Many but not all cuckoo species are parasitic [19,20]. Most parasitic cuckoo species are present in the Old-World tropics, whereas species diversity of parasitic cuckoos is very low in the Northern Hemisphere temperate regions [21]. Temperate zone cuckoos are long-distance migrants wintering in tropical regions [14,18]. Temperate cuckoos are a good model system to study host–parasite and predator–prey phenological shifts because they are highly detectable in the field due to their very distinct vocalization and their arrival can be compared with those of other migratory species, especially passerines, some of which are parasitized by cuckoos [14,22,23]. Moreover, cuckoos are specialized predators feeding mainly on easily detectable distinct prey, such as hairy caterpillars and large beetles. The phenology of this prey may have an important influence on the population dynamics of cuckoos [18,19,24]. Climate-driven spring arrival shifts and potential phenological mismatch in arrival dates between cuckoos and their hosts were previously described from regions with a single cuckoo species, particularly in Europe [14,15]. Although many world regions host multiple brood parasitic and host species [21], we are unaware of any study simultaneously comparing the arrival dates of multiple cuckoo species with those of their hosts and the emergence time of their primary prey.

Here, we focused on the changes in arrival phenology of two sympatric and interacting cuckoos, common cuckoo *Cuculus canorus* and oriental cuckoo *C*. *optatus*, and 14 avian species recognized as cuckoo hosts, as well as emergence dates of their two main prey types (hairy caterpillars and *Melolontha hippocastani* beetles) over 36 years (1988–2023) in Tatarstan (Russia). Both cuckoo species are long-distance migrants wintering in tropical regions (sub-Saharan Africa, and southeast Asia and Australia, respectively) with declining populations [18,25]. Both cuckoos spatially overlap in the Kazan region and inhabit coniferous and deciduous forests and woodlands [26], although oriental cuckoos prefer more habitats with spruce *Picea sibirica* and fir *Abies* spp. stands in the studied region (O. Askeyev 1988–2023, personal observation). Common and oriental cuckoos share several host species (although oriental cuckoo parasitizes mainly *Phylloscopus* spp. warblers) [18] and aggressive (including physical) interactions between them can be frequent in the Kazan region (O. Askeyev, personal observation, 1988–2023). We (i) document changes in the arrival timing of the two cuckoos and their host species, and the emergence date of cuckoo prey over time, and (ii) examine correlates of those timings and temporal trends in a potential phenological mismatch in arrival time of two cuckoos and their hosts as well as emergence time of their prey. Multispecies comparison and analysis of observations from non-European regions may help us to examine the generality of previous findings.

## 2. Material and methods

### 2.1. Study area

Field observations were carried out by skilled ornithologists (A.O.A., O.V.A. and I.V.A.) with long-term experience in the Kazan region, Tatarstan Republic, Russia, during the period 1988–2023. The sampling plot covers an area of approximately 1200 km^2^, centred around the city of Kazan (55°45′ N, 49°08′ E) (electronic supplementary material, figure S1) and includes various habitats, such as sub-taiga coniferous–deciduous mixed forests, farmlands, rivers, lakes and human settlements. The area has a continental climate with a mean annual temperature of 3.6°C (mean monthly range: −12.1°C in January to 19.4°C in July). Mean annual precipitation is approximately 530 mm, and snow cover lasts 141–164 days. Our fieldwork was designed to monitor arrival phenology in all included species simultaneously. Visual and acoustic field monitoring was made several times weekly during the spring and the sampling effort was relatively constant over the study period (see also [27,28]). Specifically, the sampling area included five transects (approx. 70 km in total) on which phenological observations were collected four to five times on all transects each week during the whole study period. Observations were also made during the days with adverse weather. Monitoring typically included at least two observers who visited each transect sequentially on the same day and had to agree on the arrival time of the species. This approach was adapted to increase confidence in the estimates of arrival dates and decrease the possibility of interannual variation caused by sampling effort. However, we acknowledge that the large study area size, the limited number of observers and the simultaneous recording of numerous species might decrease our precision in estimating arrival dates. The observation trials were designed to monitor changes in phenology and population size (for some species) in the whole ecosystem.

### 2.2. Arrival phenology, prey emergence and arrival asynchrony

The information on the first arrival date of two cuckoo species and their potential hosts was collected directly in the field and in habitats where the two species of cuckoo co-occurred (see also [10,27–29]). We paid particular attention to vocally active cuckoos and their hosts, indicating their territorial behaviour and the start of the breeding. Moreover, cuckoos and their hosts migrate mainly during the night, often inconspicuously [30,31]. Hence, we assumed that diurnally active and singing individuals belonged to the local population. We also collected information on the first emergence dates of the primary prey type of cuckoos, i.e. hairy caterpillars and a large beetle species, the forest cockchafer *M. hippocastani* [18], in the studied region. We focused on the first arrival/emergence date rather than the mean/median date because the first arrival/emergence date is most frequently reported in the literature, making our results easily comparable to the previous ones (e.g. [4,14]). Potential host and prey species were selected based on local field observations and literature surveys (e.g. [18,32,33]). For each year, bird arrival and prey emergence dates were transformed into days after 1 January (i.e. day 1 = 1 January) prior to analysis. The relative annual advancement in dates of the arrival of cuckoo species, their hosts and prey emergence was estimated by calculating the difference between the arrival date of each cuckoo species and the arrival/emergence date of their host and prey species, respectively [14,34]; we used this as an index of potential temporal phenological asynchrony/mismatch. If cuckoos would show smaller advancement in their arrival than their hosts, they may miss the opportunity to check thoroughly territories occupied by potential hosts and lay parasitic eggs because the optimal time for parasitation is very short [35]. Moreover, a smaller rate of change in the arrival dates of cuckoos than in the emergence date of their prey may cause cuckoos to miss the peak in their prey availability [36]. Hence, we interpreted smaller advancements in cuckoo arrival dates when compared with those of their hosts and prey emergence as signs of potentially increasing phenological asynchrony/mismatch.

### 2.3. Predictor variables

We obtained data on mean monthly air temperatures in April for the period 1988–2023 from the Kazan (Opornaya) meteorological station (55°26' N, 49°08' E). All measurements were made according to World Meteorological Organization (WMO) standards. We used April temperatures because the mean arrival date of host species fell within April (median Julian date = 119.6 days; mean ± s.d. = 117.4 ± 12.2; range = 89.5–132.0) and also both cuckoos tend to arrive in the study region around the end of April (common cuckoo: median Julian date = 120 days; mean ± s.d. = 119.6 ± 3.7; range = 112–129; oriental cuckoo: median Julian date = 126 days; mean ± s.d. = 125.6 ± 4.0; range = 118–134); hence, we assumed that shifts on arrival phenology of both cuckoos and their hosts should be associated primarily with changes in April temperatures (electronic supplementary material, table S1). March is typically still cold in Kazan (mean temperature in 1988–2023 = −3.2°C), whereas May is already quite a hot month (13.9°C) in comparison with April (5.9°C).

Population density can impact the detection probability of bird arrival date because there is a higher probability of observing earlier arrival when the population is larger [37]. Hence, we also estimated mean observed densities (number of individuals per km^2^) for each cuckoo species throughout the breeding season (May–June) and for each year. Cuckoo density was estimated during the morning counts according to Ravkin's transect methods [38] without a fixed strip width with subsequent density conversion using group mean detection ranges. Cuckoo density was then estimated based on the mean bird detection distances. However, we consider our sampling effort inadequate for precise estimates due to the large size of the sampled area and small number of observers.

### 2.4. Statistical analyses

We first checked for outliers in arrival dates and asynchrony values and excluded all values falling outside the 1.5 times interquartile range above the third quartile or below the first quartile, respectively. Second, we explored correlations between continuous predictors, revealing a very strong correlation between the first emergence day for *M. hippocastani* and hairy caterpillars (*r*_Pearson_ = 0.89); other continuous predictors showed collinearity less than 0.7 (electronic supplementary material, figure S2). Hence, in all regression analyses where we used prey emergence date as a predictor, we ran two alternative models, either with the emergence date of hairy caterpillars or *M. hippocastani* included. We also used log_10_-transformed cuckoo density values because raw values were strongly right-skewed, and each continuous predictor was centred and scaled around the mean using the *scale* function.

First, we explored environmental variables correlated with the arrival date of cuckoos and their potential avian hosts and the emergence date of cuckoo prey. We modelled the arrival date of cuckoos (response variable) as a function of year, cuckoo species (common cuckoo/oriental cuckoo), prey emergence date, mean April temperature and cuckoo density using linear models using a Gaussian family. Adjusted deviance explained by the models was calculated using the *Dsquared* function in the modEvA package [39]. Model assumptions were visually checked using the *plot* function. Next, we modelled the arrival date of cuckoos' host species (response variable) as a function of year, prey emergence date and mean April temperature while controlling for host species identity (random intercept; to control for non-independence of multiple entries for the same host species) using linear mixed models with Gaussian error distribution and fitted by maximum likelihood. In contrast to cuckoos, most cuckoo hosts do not feed on hairy caterpillars or large beetles; however, we included the emergence date for these prey species in the analysis because they may indicate the emergence date for insect prey which is eaten by avian host species. We calculated variances explained by the fixed and random effects (conditional *R*^2^) and by the fixed effects only (marginal *R*^2^) using the *r.squaredGLMM* function in the MuMIn package [40]. Finally, we modelled prey emergence date as a function of year, temperature, prey species (hairy caterpillars or *M. hippocastani*) and interaction between year and prey species using linear models using a Gaussian family. In all full models, we again checked for the outliers and also the degree of multicollinearity among all continuous and categorical predictors by calculating variation inflation factors (VIFs) using the *vif* function in the car package [41]. In general, VIFs > 4 may indicate problematic collinearity [42]; we detected VIF > 4 for several predictors when modelling the arrival dates of cuckoos. Hence, we simplified our main models and generated the best candidate models set using an automatic backward stepwise model selection using the *dredge* function in the MUMIn package [43]. The final inference was then drawn from a single best model with the lowest AICc (Akaike's information criterion with correction for small sample size) values (ΔAICc < 2). It is assumed that models with AICc values differing by less than 2 had similar statistical support [44] but because alternative best models typically provided very similar results to a single best model, we provide only results of a single best model in the main text and results of alternative best models (i.e. those with ΔAICc < 2 from a single best model) in the electronic supplementary material. We did not use model simplification in models for hosts and prey emergence because all predictors were significantly associated with the arrival date in the main model, or we did not detect problematic collinearity, respectively.

We then modelled the association between arrival asynchrony between cuckoos and each of their hosts (response variable) and year, cuckoo species (common cuckoo and oriental cuckoo, respectively), prey emergence date (each model used either data for hairy caterpillars or *M. hippocastani*), temperature and cuckoo density for each species while controlling for host species identity (random intercept) using linear mixed models with Gaussian error distribution and fitted by maximum likelihood. We also modelled the association between arrival asynchrony between cuckoos and their prey species (either hairy caterpillars or *M*. *hippocastani*), and year, temperature, cuckoo density and cuckoo species, and interaction between year and cuckoo species using a linear model with Gaussian error distribution. In both cases, we simplified our main models because of the collinearity between predictors using an automatic model selection. In all full models, we checked the models' assumptions by plotting the residuals using the *simulateResiduals* function (999 simulations) in the DHARMa package [45] revealing no major violations. Finally, we tested for correlation in arrival dates between the two cuckoo species using Pearson correlation coefficients. We tested whether one of the cuckoo species arrived earlier than the other one using a paired two-sided *t*-test. All statistical analyses were conducted in R v. 4.1.2 [46].

## 3. Results

The arrival date of the two cuckoo species in Tatarstan was strongly correlated across the years (*r*_Pearson_ = 0.88, *t* = 10.993, d.f. = 34, *p*-value = <0.001), although oriental cuckoo arrived on average later than common cuckoo (paired *t*-test: *t* = −19.158, d.f. = 35, *p*-value < 0.001). The arrival date of both cuckoos and their potential host species was negatively associated with year and also with April temperature (electronic supplementary material, figures S3–S4; tables S1–S4). In other words, cuckoos and their hosts arrive earlier from wintering to breeding grounds in more recent years and years with hotter weather in April. However, the arrival date of cuckoo hosts was also positively correlated with the emergence day of the insect prey. We also found that prey emerged earlier in more recent years and that prey emergence was strongly linked to April temperature (electronic supplementary material, table S5; and figure S5). Finally, April temperature significantly increased in the recent years, although the correlation was weak (electronic supplementary material, figure S2).

**Figure 1.**
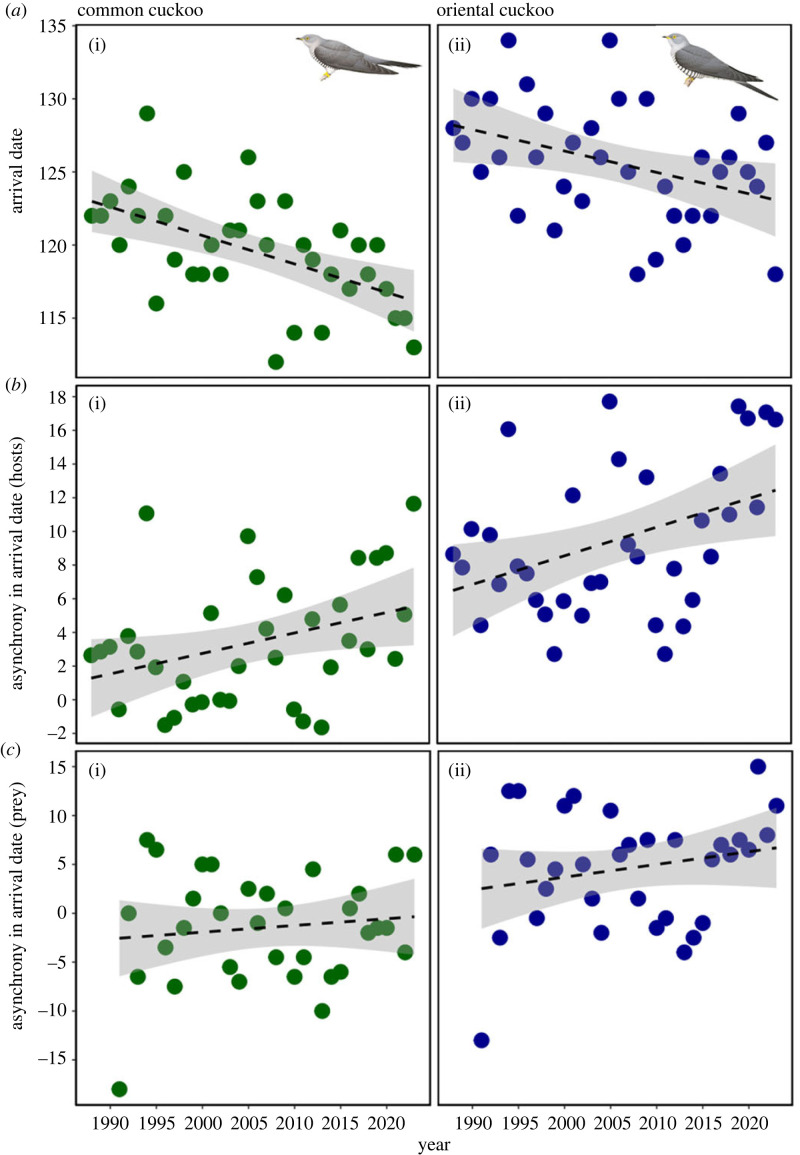
Changes in (*a*) the first arrival date of two cuckoo species (*a*(i),*b*(i),*c*(i)/green: common cuckoo *Cuculus canorus*; *a*(ii),*b*(ii),*c*(ii)/blue: oriental cuckoo *C*. *optatus*), and mean relative advancement (asynchrony) in arrival date between cuckoos and (*b*) their hosts or (*c*) prey (i.e. hairy caterpillars and *Melolontha hippocastani*), respectively, during the 1988–2023 period. Dots: values of first arrival date and mean asynchrony for each year and cuckoo species; lines: simple linear model outputs within each figure panel; shaded regions: 95% confidence intervals for each section. Bird illustrations reproduced with permission from Handbook of the Birds of the World ©, Lynx Edicions.

**Figure 2.**
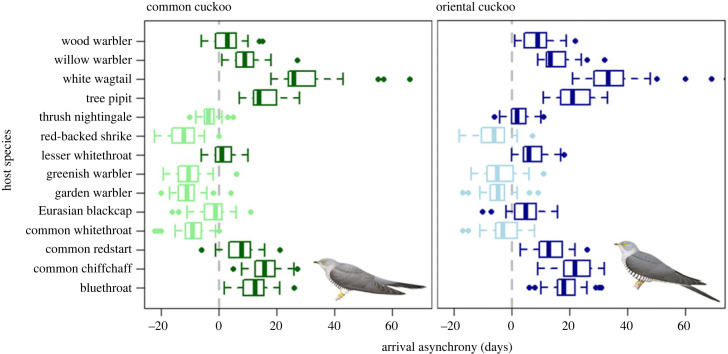
Relative arrival advancement (asynchrony) between common (green) *Cuculus canorus* and oriental (blue) cuckoo *C*. *optatus* and their host species. Positive values and dark colours indicate that cuckoos arrive later to the breeding grounds than their hosts, negative values and light colours indicate that cuckoo arrival precedes that of their hosts. Box plots show the median, upper (75%) and lower (25%) quartiles (vertical lines in the middle, right and left of the box, respectively), 1.5 times the interquartile range (whiskers) and outliers (dots). Bird illustrations reproduced with permission from Handbook of the Birds of the World ©, Lynx Edicions.

The arrival date in cuckoos advanced significantly less than in their hosts from 1988 to 2023 (figure 1), with both cuckoo species usually arriving later than their hosts (figure 2). The potential asynchrony was generally higher for oriental cuckoo than common cuckoo, but asynchrony increased over the years with a similar slope in both cuckoo species (table 1, electronic supplementary material, tables S6 and S7). Moreover, the oriental cuckoo that parasitizes mainly *Phylloscopus* spp. warblers consistently showed smaller advancements in arrival date than its major hosts (electronic supplementary material, figure S7). Across all years, we found that common cuckoos arrived approximately at the same time as the first emergence of their prey was reported (mean date difference ± s.d. = −1.5 ± 5.6 days) whereas oriental cuckoos arrived on average almost a week later (mean ± s.d. = 4.6 ± 6.0 days). The cuckoos’ arrival timing also advanced less than the emergence date of their prey from 1988 to 2023 and again with similar slopes in both cuckoo species (table 2, electronic supplementary material, tables S8 and S9).

**Table 1.** Results of a single best multiple linear mixed regression testing association between arrival asynchrony of the two cuckoo species (common cuckoo *Cuculus canorus* and oriental cuckoo *C*. *optatus*) and their hosts, and a set of predictors, including the date of prey emergence (hairy caterpillars), mean April air temperature (°C), density of each cuckoo species in each year (individuals/km^2^), year and cuckoo species, and interaction between them, while controlling for host species identity, between 1988 and 2023 in Tatarstan (Russia). Conditional *R*^2^ of the model = 0.85; marginal *R*^2^ = 0.061. Variance of the random intercept (Species) ± s.d. = 132.86 ± 11.53. For each predictor, we report slope (estimate) and its standard error (s.e.), degrees of freedom (d.f.), *t*-values and *p*-values. Statistically significant associations are highlighted in italics. For results of full and alternative best-simplified models (with ΔAICc < 2), see electronic supplementary material, tables S6 and S7.

variable	estimate	s.e.	d.f.	*t*-value	*p*-value
intercept	3.215	3.089	14.074	1.041	0.316
temperature	0.293	0.164	984.999	1.787	0.074
*year*	*1.158*	*0.164*	*985.011*	*7.064*	*<0.001*
*cuckoo species (optatus)*	*5.956*	*0.324*	*984.998*	*18.370*	*<0.001*

**Table 2.** Results of a single best multiple linear regression testing association between arrival asynchrony of the two cuckoo species (common cuckoo *Cuculus canorus* and oriental cuckoo *C*. *optatus*) and emergence date of their prey (hairy caterpillars), and a set of predictors, including mean April air temperature (°C), density of each cuckoo species in each year (individuals/km^2^), year and cuckoo species, and interaction between them, in Tatarstan (Russia), between 1988 and 2023. Adjusted *R*^2^ of the model = 0.41; *F*_3,61_ = 16.11, For each predictor, we report slope (estimate) and its standard error (s.e.), *t*-values and *p*-values. Statistically significant associations are highlighted in italics. For results of models with hairy caterpillars replaced by *Melolontha hippocastani*, and simplified alternative best models, see electronic supplementary material, tables S8 and S9.

variable	estimate	s.e.	*t*-value	*p*-value
intercept	3.031	0.558	5.435	<0.001
*temperature*	*2.036*	*0.566*	*3.597*	*<0.001*
*cuckoo density*	*−3.577*	*0.613*	*−5.835*	*<0.001*
*year*	*1.521*	*0.617*	*2.466*	*0.016*

## 4. Discussion

Our results for two sympatric parasitic and migratory cuckoo species, common and oriental cuckoo, in Tatarstan (Russia), showed that climate change may not only cause shifts in the arrival date of cuckoos, their hosts and prey emergence but may also increase phenological asynchrony between these groups. We found that both cuckoo species have advanced their spring arrival to the breeding grounds over the sampled 36 year period. However, cuckoo arrival advancement was smaller than in most hosts, potentially causing increasing temporal mismatch between cuckoos and their hosts in breeding cycles over time. Moreover, cuckoo arrival advancement was also slightly slower than shifts in prey emergence over time. This may indicate that these brood parasites cannot track both host and prey phenological changes. More generally, our results suggest that the effects of climate change may have substantially adverse effects on species with closely interlinked life cycles such as in the case of parasitic or symbiotic interactions or prey specialists.

Arrival and breeding dates of many migratory birds have advanced during the last decades [10,47,48] as has emergence date of insect prey of birds [49,50]. We found that arrival dates in both cuckoo species were strongly correlated, indicating that similar abiotic and biotic factors may mandate their spring migration. Previous research showed that the arrival phenology of birds wintering in geographically distant world regions may be affected by local weather conditions [51]. Arrival dates of cuckoos, their hosts and prey were negatively correlated with April temperatures at cuckoos, breeding grounds. Spring arrival of common cuckoos is strongly determined by environmental conditions in their wintering grounds [44]; however, we could not test this directly in our study system because information on local cuckoos' population migratory routes and wintering grounds is lacking. Cuckoo arrival is highly synchronous within a population but still may be under endogenous control, suggesting that various ecological and physiological constraints limit variation in the timing of this event in cuckoos [52,53]. As a result, we found evidence that differential shifts in arrival dates between cuckoos and their hosts and date of prey emergence may lead to increased temporal mismatch with their host and prey spring phenology, which may have serious consequences on cuckoo, host and prey fitness and population dynamics.

Temporal asynchrony in cuckoo-host arrival may result from decoupling departure signals between wintering and breeding grounds. The onset of migration in long-distance migrants may be controlled mainly by internal mechanisms [54–57] but birds can also use external environmental cues in wintering grounds and along migration routes to adjust migration timing accordingly [52,57–59]. However, the observed temporal asynchrony suggests that the cuckoo's ability to follow changing climate is constrained. The weaker temporal response of cuckoos to climatic patterns than in their hosts and prey may reflect a smaller evolutionary ability to respond to selection for earlier arrival, or restricted phenotypic plasticity [60–62]. For example, long-distance migrants including cuckoos wintering in more distant regions may be less able to predict conditions on the breeding grounds than shorter-distance migrants (including several host species included in this study) or may predominantly rely more on stable and constant (circannual and endogenous) mechanisms to time their migration such as changes in the day length rather than temperature and other environmental cues impacted more by climate change [16,17]. As a result, migratory parasitic cuckoos may miss the optimal timing of parasitizing their hosts or peak in prey availability [14,63].

Common and oriental cuckoos have declining populations [18,25]. The causes of these declines are unclear but our results indicate a possible link to the climate-driven increase in temporal asynchrony in breeding phenology between cuckoos, their hosts [14,15] (but see [22]) but also prey emergence. For example, previous research showed that short-distance migratory hosts have advanced their arrival time more than long-distance migrants, including common cuckoo [14,48]; hence, cuckoos may be increasingly missing breeding opportunities because of an increasing delay in their arrival [14]. Cuckoos of genus *Cuculus* are also dietary specialists, feeding mainly on hairy caterpillars and large beetles [18], and cuckoos seem to be declining in regions with declining prey densities [64]. Climate change may also increase spatial mismatch between cuckoos and their hosts [65] or prey [66,67]. For example, migratory birds may be particularly vulnerable to mismatches in the resource availability at breeding grounds rather than a mismatch in migratory host availability because climate change effects are not equal over the globe [68]; wintering grounds and migratory routes of cuckoos and their hosts may overlap and migratory behaviour of the two groups may be affected by similar environmental cues. However, our results showed stronger potential asynchrony between cuckoos and their hosts than with their prey at breeding grounds (figure 1*b,c*). Finally, we found that the mean local population densities of both cuckoo species are declining with a similar slope in the sampled area (electronic supplementary material, table S10). This indicates that although both cuckoo species may compete for their hosts, potentially disadvantaging later arriving oriental cuckoo, phenological asynchrony and potential mismatch may have a similar effect on populations of both species.

Cuckoos are often host specialists, with at least some species even comprising female host-specific subpopulations specialized in parasitizing different host species [69,70]. Hence, temporal and spatial mismatch may cause host switch and/or change the relative proportion of different host-specific subpopulations in cuckoos and subsequently change parasitism rates in host populations [15]; host species, particularly those arriving earlier, may take an advantage of climate-driven arrival mismatch to escape parasitation by cuckoos whereas others such as late-arrivals may suffer from higher rates of brood parasitism. Cuckoos as prey specialists may also potentially contribute to the top-down control of populations of Lepidoptera species with hairy larvae, including several important crop pests, and affect their population dynamics [71].

In conclusion, we provide the first evidence that climate change might disrupt the association between the life cycles of migratory cuckoo species and their hosts and prey, respectively. We have shown that increasing phenological asynchrony in arrival date between cuckoos and their hosts is also present (a) in non-European parts of the common cuckoo range, and (b) in other migratory cuckoo species (oriental cuckoo). Moreover, climate change may also disrupt trophic interactions between cuckoos and their prey.

## Data Availability

The data are provided in the electronic supplementary material [72].
